# Beyond Neighborhood Boundaries: GPS-Based Activity Space, Built Environment, and Cognitive Health

**DOI:** 10.1093/geroni/igaf122.949

**Published:** 2025-12-31

**Authors:** Jinshil Hyun, Nelson Roque, Gina Lovasi, Dean Hosgood, Mindy Katz, Richard Lipton, Charles Hall

**Affiliations:** Albert Einstein College of Medicine, Bronx, New York, United States; The Pennsylvania State University, University Park, Pennsylvania, United States; Drexel University, Philadelphia, Pennsylvania, United States; Albert Einstein College of Medicine, Bronx, New York, United States; Albert Einstein College of Medicine, Bronx, New York, United States; Albert Einstein College of Medicine, Bronx, New York, United States; Albert Einstein College of Medicine, Bronx, New York, United States

## Abstract

Neighborhood effects on late-life cognition have predominantly focused on built environment features within residential Census Tracts. However, individuals are active agents who, guided by personal goals and preferences, navigate environments beyond their residential context. GPS technology allows us to more accurately capture individuals’ true spatiotemporal exposure to built environment features relevant for cognitive, social, and physical activities. Here, we compare how built environment features within residential Census tracts versus person-specific GPS-derived activity spaces are associated with late-life cognitive outcomes. Participants (N = 126) were from Bronx, NY in the Einstein Aging Study (mean age=76; 63% females; 37% non-Hispanic Blacks). Person-level activity space was assessed using minimum convex hull areas derived from GPS data collected over a 14-day period. Five cognitive domains (episodic memory, language, executive function, processing speed, visuospatial) were derived by averaging a subset of scores from 13 standard neurocognitive tests, with final scores in T-score units. Results from linear regression suggested that a higher availability of built environment features supporting physical and cognitive activities within Census tracts were associated with better speed (p=.032), visuospatial (p = 0.012), and executive function (p = 0.010). Person-specific activity spaces showed more consistent associations; individuals whose daily environments provided greater opportunities for physical, social, and cognitive activities had better cognitive performance in episodic memory (ps=.000∼.018), language (ps=.001∼.043), executive function (ps=.000∼.005), and processing speed (ps=.035∼.038). Person-specific activity space measures may more accurately reflect individuals’ true exposure to neighborhood environments that influence cognitive health. Further investigation into behavioral mechanisms is warranted to identify potential targets for cognitive health interventions.

